# Mathematical Model and Analysis on the Impact of Awareness Campaign and Asymptomatic Human Immigrants in the Transmission of COVID-19

**DOI:** 10.1155/2022/6260262

**Published:** 2022-05-28

**Authors:** Alemzewde Ayalew Anteneh, Yezbalem Molla Bazezew, Shanmugasundaram Palanisamy

**Affiliations:** ^1^Department of Mathematics, Hawassa University, Ethiopia; ^2^Department of Mathematics, Mizan Tepi University, Ethiopia

## Abstract

In this study, an autonomous type deterministic nonlinear mathematical model that explains the transmission dynamics of COVID-19 is proposed and analyzed by considering awareness campaign between humans and infectives of COVID-19 asymptomatic human immigrants. Unlike some of other previous model studies about this disease, we have taken into account the impact of awareness c between humans and infectives of COVID-19 asymptomatic human immigrants on COVID-19 transmission. The existence and uniqueness of model solutions are proved using the fundamental existence and uniqueness theorem. We also showed positivity and the invariant region of the model system with initial conditions in a certain meaningful set. The model exhibits two equilibria: disease (COVID-19) free and COVID-19 persistent equilibrium points and also the basic reproduction number, *R*_0_ which is derived via the help of next generation approach. Our analytical analysis showed that disease-free equilibrium point is obtained only in the absence of asymptomatic COVID-19 human immigrants and disease (COVID-19) in the population. Moreover, local stability of disease-free equilibrium point is verified via the help of Jacobian and Hurwitz criteria, and the global stability is verified using Castillo-Chavez and Song approach. The disease-free equilibrium point is both locally and globally asymptotically stable whenever *R*_0_ < 1, so that disease dies out in the population. If  *R*_0_ > 1, then disease-free equilibrium point is unstable while the endemic equilibrium point exists and stable, which implies the disease persist and reinvasion will occur within a population. Furthermore, sensitivity analysis of the basic reproduction number, *R*_0_ with respect to model parameters, is computed to identify the most influential parameters in transmission as well as in the control of COVID-19. Finally, some numerical simulations are illustrated to verify the theoretical results of the model.

## 1. Introduction

Coronaviruses are a large family of viruses that may cause illness in animals and humans. In humans, several coronaviruses are known to cause respiratory infections ranging from the common cold to more severe diseases such as Middle East respiratory syndrome (MERS) and severe acute respiratory syndrome (SARS). The most recently discovered human coronavirus disease which is caused by a novel coronavirus severe acute respiratory syndrome 2 (SARS-CoV-2) is coronavirus disease 2019 (COVID-19) [1, 2]. This disease was first identified in December 2019 in Wuhan, China, with common manifestation appears to be pneumonia [3–5]. One of the things which makes this new virus so dangerous is that it spreads very quickly between peoples. COVID-19 is transmitted from human to human via direct contact with contaminated surfaces and through respiratory droplets' inhalation from infected individuals [4, 6, 7]. The most common symptoms of COVID-19 include the following: fever, dry cough, shortness of breath, and fatigue [3, 8]. Other fewer symptoms may include the following: sore throat, headache, chills, congestion, nausea, and diarrhea. The incubation period (time from exposure to the development of symptoms) of COVID-19 is somewhere between 2 and 14 days, on average around five days after exposure to the virus [9, 10].

The World Health Organization (WHO) declared the outbreak as a Public Health Emergency of International Concern on January 2020 and a pandemic on 11 March 2020 [11]. In China mainland until March 8, 2020, a total of 80,868 confirmed cases and 3,101 deaths were recorded due to COVID-19 [3]. The outbreak of COVID-19 has spread to 222 countries and territories, inflicted more than 116.17 million confirmed cases, and claimed more than 2.58 million lives, as reported on March 7th, 2021 [12]. Globally, over 3.1 million new cases and just over 54,000 new deaths were reported during the week of 27 September to 3 October 2021 and more than 197 million new cases, and among these, more than 4 million individuals have died up to the end of July 2021 [13, 14].

Mathematical model is a mathematical equation that describes changes in the system with time, and it is useful to accurately predict the evolution of infectious disease (COVID-19), and this in turn helps to give an insight for health workers and government to deal on the most influential parameters in COVID-19 transmission [15]. Since COVID-19 outbreak, different scholars studied the transmission dynamics of the disease (COVID-19) by considering different scenarios [1, 2, 5, 9–11, 14–21] to curb its spread with the help of epidemiological mathematical model. However, all the above studies failed to consider the impact of awareness campaign and influx of COVID-19 asymptomatic human immigrants in the control of COVID-19 transmission. In this study, we inspire to modify the model [16] to fulfill the entire gap. Our proposed model is different from others, and it is that the class of susceptible human population is subdivided into two: individuals who have awareness about COVID-19 and apply all the recommended mitigation of COVID-19 to save themselves and others are classified as aware susceptible human population class, denoted by *A*_*h*_ and individuals who have no awareness about COVID-19 or even they have awareness but they do not give an emphasis for the severity of COVID-19 are classified as unaware susceptible human population class, denoted by *U*_*h*_. The rate of COVID-19 asymptomatic human immigrants is also included. The remaining part of this study is structured as follows: in Section 2, we developed our mathematical model for the transmission of COVID-19 dynamics. In Section 3, the analytical analysis of the model established. In Section 4, numerical simulations of the model are illustrated. The conclusion and concluding remark with suggestions are provided in Sections 5 and 6.

## 2. Model Formulation

**Figure 1:**
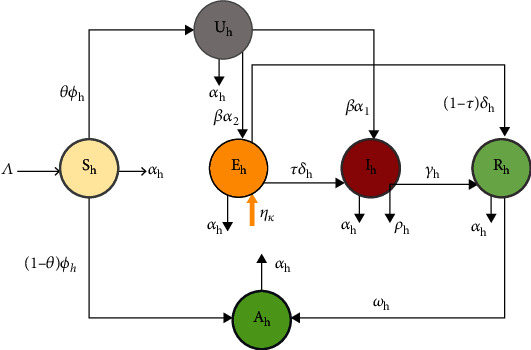
Flow diagram of COVID-19 model.

In this section, the total number of human population at a given time *t* is denoted by *N*_*h*_(*t*) and it is catagorized into six subclasses named as susceptible human population class (*S*_*h*_), aware susceptible human population class (*A*_*h*_), unaware susceptible human population class (*U*_*h*_), exposed human population class (*E*_*h*_), infectious human population class (*I*_*h*_), and recovered human population class (*R*_*h*_). The class of aware susceptible human will increase depending on the movement of susceptible human population and recovered human population class due to awareness created. Unaware susceptible human population class increases due to the movement of susceptible human population class and decreases due to contact with infectives of COVID-19 humans and progress into exposed and infectious human population class. In the formulation of our model, the following additional assumptions are important:

1. It is assumed that all recruited humans either by birth or immigration into susceptible human class are not carriers of COVID-19
2. We consider only immigrants of COVID-19 asymptomatic human in our model with a rate of *η*_*h*_
3. It is assumed that the influx rate *η*_*h*_ of COVID-19 asmptomatic human immigrants is not constant
4. It is assumed that the class of unaware susceptible human populations becomes infective if they contact with exposed or infectious individuals at the rate *β* with probability of *α*_2_ and *α*_1_, respectively
5. It is assumed that the rate of asymptomatic human immigrants *η*_*h*_ is less than the progression rate of exposed human into infectious or recovered human class *δ*_*h*_
6. It is assumed that a proportion of *θ* of susceptible population properly apply COVID-19 mitigation measures and hence progress into aware susceptible human class and the remaining proportion (1−*θ*) move to unaware susceptible human class by the rate of *ϕ*_*h*_
7. It is assumed that recovered human populations develop permanent immunity due to their improved immunity or successful hospitalized treatment, and they join the aware susceptible human population class at the rate of  *ω*_*h*_
8. It is assumed that all parameters involved in the model are nonnegative. With regard to the above considerations, the compartmental flow diagram is shown below in Figure 1

Based on assumptions and the flow diagram of COVID-19 dynamics (Figure 1), Table 1 the model is governed by the following system of autonomous type nonlinear ordinary differential equations:

(1) $$\begin{array}{c} \left\{\begin{array}{c} \frac{dS_{h}}{dt}=\Lambda_{h}-\left(\alpha_{h}+\phi_{h}\right)S_{h},\\ \frac{dA_{h}}{dt}=\left(1-\theta \right)\phi_{h}S_{h}-\alpha_{h}A_{h}+\omega_{h}R_{h},\\ \frac{\;dU_{h}}{dt}=\theta \phi_{h}S_{h}-\beta \left(\alpha_{2}E_{h}+\alpha_{1}I_{h}\right)U_{h}-\alpha_{h}U_{h},\\ \frac{dE_{h}}{dt}=\beta \left(\alpha_{2}E_{h}+\alpha_{1}I_{h}\right)U_{h}-\left(\alpha_{h}+\delta_{h}-\eta_{h}\right)E_{h},\\ \frac{\;dI_{h}}{dt}=\tau \delta_{h}E_{h}-\left(\alpha_{h}+\rho_{h}+\gamma_{h}\right)I_{h},\\ \frac{dR_{h}}{dt}=\left(1-\tau \right)\delta_{h}E_{h}+\gamma_{h}I_{h}-\left(\alpha_{h}+\omega_{h}\right)R_{h}, \end{array}\right. \end{array}$$

with initial conditions: *S*_*h*(0)_ > 0, *A*_*h*(0)_ ≥ 0, *U*_*h*(0)_,  *E*_*h*(0)_ ≥ 0 , *I*_*h*(0)_ ≥ 0 , *R*_*h*(0)_ ≥ 0, and 0 ≤ *θ* ≤ 1, 0 ≤ *τ* ≤ 1.

## 3. Model Analysis

The validity and the authenticity of any mathematical model depend on the existence and uniqueness of its solutions. In this section, the basic properties of the model system of equations (1) include the following: the existence and uniqueness of solutions, positivity of solutions, invariant region, and basic reproduction number, and equilibria with their stability analysis are focused.

**Lemma 1 (Existence and Uniqueness of Solutions).**

If the initial data be (*S*_*h*_(0) > 0, *A*_*h*_(0) ≥ 0, *U*_*h*_(0) ≥ 0, *E*_*h*_(0) ≥ 0, *I*_*h*_(0) ≥ 0, *R*_*h*_(0) ≥ 0) to the system equation (1), then there exists a unique solution in *C*(ℝ^+^, ℝ_+_^6^), ∀*t* ≥ 0.

**Proof**

The model system of equation (1) can be rewritten in the form of $\dot{x}=f\left(x\right)$, where

(2) $$\begin{array}{c} \dot{x}=\left[\begin{array}{c} {\dot{S}}_{h}\\ {\dot{A}}_{h}\\ {\dot{U}}_{h}\\ {\dot{E}}_{h}\\ {\dot{I}}_{h}\\ {\dot{R}}_{h} \end{array}\right]\;f\left(x\right)=\left[\begin{array}{c} \Lambda_{h}-\left(\alpha_{h}+\phi_{h}\right)S_{h}\\ \left(1-\theta \right)\phi_{h}S_{h}-\alpha_{h}A_{h}+\omega_{h}R_{h}\\ \;\theta \phi_{h}S_{h}-\beta \left(\alpha_{2}E_{h}+\alpha_{1}I_{h}\right)U_{h}-\alpha_{h}U_{h}\\ \;\beta \left(\alpha_{2}E_{h}+\alpha_{1}I_{h}\right)U_{h}-\left(\alpha_{h}+\delta_{h}-\eta_{h}\;\right)E_{h}\\ \tau \delta_{h}E_{h}-\left(\alpha_{h}+\rho_{h}+\gamma_{h}\;\right)I_{h}\\ \;\left(1-\tau \right)\delta_{h}E_{h}+\gamma_{h}I_{h}-\left(\alpha_{h}+\omega_{h}\;\right)R_{h} \end{array}\right]. \end{array}$$

Each of the right hand side components of *f* (*x*) in system [2] is continuously differentiable almost everywhere in *C*(ℝ^+^, ℝ_+_^6^), which implies *f* is locally Lipchitz (f ∈ *c*^1^). Hence, by the fundamental existence and uniqueness theorem [22], the model system of equation (1) exhibits a unique solution locally in  ℝ_+_^6^ for all time *t* ≥ 0. Since the model system of equations [1] monitors human population, it is necessary to show that all its solutions must be nonnegative for future time *t*. This will be established by the following theorem.

**Theorem 1 (Positivity of Model Solutions).**

If the initial condition be *Σ* = {*S*_*h*_(0) > 0, *A*_*h*_(0) ≥ 0, *U*_*h*_(0) ≥ 0, *E*_*h*_(0) ≥ 0, *I*_*h*_(0) ≥ 0, *R*_*h*_(0) ≥ 0}, then the solution set {*S*_*h*_(*t*), *A*_*h*_(*t*), *U*_*h*_(*t*), *E*_*h*_(*t*), *I*_*h*_(*t*), *R*_*h*_(*t*)} of model (1) is positively invariant for all time *t* ≥ 0.

**Proof**

From the model system of equation (1), consider the first equation:

*dS*_*h*_/*dt* = *Λ*_*h*_ − (*α*_*h*_ + *ϕ*_*h*_)*S*_*h*_; then, by integrating factor method, the solution becomes the following:

(3) $$\begin{array}{c} S_{h}\left(t\right)=\Lambda_{h}/\alpha_{h}+\phi_{h}+\left(S_{h}\left(0\right)-\Lambda_{h}/\alpha_{h}+\phi_{h}\right)e^{-\left(\alpha_{h}+\phi_{h}\right)\;t}>0. \end{array}$$

Hence, *S*_*h*_(*t*) > 0 for all time *t* ≥ 0. From model system of equation (1), consider the third equation:

*dU*_*h*_/*dt* = *θϕ*_*h*_*S*_*h*_ − *β*(*α*_2_*E*_*h*_ + *α*_1_*I*_*h*_)*U*_*h*_ − *α*_*h*_*U*_*h*_. Assume that for the first time *t*_1_, *U*_*h*_(*t*_1_) = 0, *dU*_*h*_/*dt* ≤ 0, and it is true that (*S*_*h*_(*t*), *A*_*h*_(*t*), *E*_*h*_(*t*), *I*_*h*_(*t*), *R*_*h*_(*t*)) > 0 for *t* ∈ (0, *t*_1_). Based on our assumption, *dU*_*h*_/*dt* = *θϕ*_*h*_*S*_*h*_, and then, integrate both sides and substitute the solution of *S*_*h*_(*t*) obtained from the above, and we get that *U*_*h*_(*t*_1_) = *θϕ*_*h*_(*Λ*_*h*_/*α*_*h*_ + *ϕ*_*h*_ + (*S*_*h*_(0) − *Λ*_*h*_/*α*_*h*_ + *ϕ*_*h*_)*e*^−(*α*_*h*_ + *ϕ*_*h*_)*t*_1_^) ≥ 0 which contradicts with our assumption. Hence, *U*_*h*_(*t*) ≥ 0, ∀*t* ≥ 0.

Similarly, it can be shown analogously that (*A*_*h*_(*t*), *E*_*h*_(*t*), *I*_*h*_(*t*), *R*_*h*_(*t*)) ≥ 0, ∀*t* ≥ 0.

Hence, all solutions of model system [1] are positive for all future time *t* ≥ 0.

**Theorem 2 (Invariant Region).**

There exists a domain *Σ* in which the solution set (*S*_*h*_(*t*), *A*_*h*_(*t*), *U*_*h*_(*t*), *E*_*h*_(*t*), *I*_*h*_(*t*), *R*_*h*_(*t*)) of model equation (1) is positively invariant.

**Proof**

The total human population size can be determined by *N*_*h*_(*t*) = *S*_*h*_(*t*) + *A*_*h*_(*t*) + *U*_*h*_(*t*) + *E*_*h*_(*t*) + *I*_*h*_(*t*)+*R*_*h*_(*t*). Then, the time derivative of *N*_*h*_(*t*) along the solutions of model system (1) gives the following:

(4) $$\begin{array}{c} \frac{{dN}_{h}}{dt}=\Lambda_{h}-\alpha_{h}N_{h}\left(t\right)-\rho_{h}I_{h}\left(t\right)+\eta_{h}E_{h}\left(t\right). \end{array}$$

In the absence of disease (COVID-19) in the population and COVID-19 asymptomatic human immigrants,

(5) $$\begin{array}{c} \frac{{dN}_{h}}{dt}\leq \Lambda_{h}-\alpha_{h}N_{h}\left(t\right)\Rightarrow N_{h}\left(t\right)=\frac{\Lambda_{h}}{\alpha_{h}\;}+\left(N_{h}\left(0\right)-\frac{\Lambda_{h}}{\alpha_{h}\;}\right)e^{-\alpha_{h}\;t}, \end{array}$$

where

(6) $$\begin{array}{c} N_{h}\left(0\right)=S_{h}\left(0\right)+A_{h}\left(0\right)+U_{h}\left(0\right)+E_{h}\left(0\right)+I_{h}\left(0\right)+R_{h}\left(0\right). \end{array}$$

Thus, if *N*_*h*_(0) ≤ *Λ*_*h*_/*α*_*h*_, then *N*_*h*_(*t*) ≤ *Λ*_*h*_/*α*_*h*_ as t⟶∞.

Therefore, *Σ* = {(*S*_*h*_(*t*), *A*_*h*_(*t*), *U*_*h*_(*t*), *E*_*h*_(*t*), *I*_*h*_(*t*), *R*_*h*_(*t*)) ∈ ℝ_+_^6^ : *N*_h_(0) ≤ *N*_h_(*t*) ≤ *Λ*_*h*_/*α*_*h*_} is the feasible solution of model equation (1) which implies the total number of human population is positively invariant; hence, each solution in the system of model equation (1) is positively invariant. Therefore, the model is biologically meaningful and mathematically well-posed in the region *Σ*.

### 3.1. COVID-19-Free Equilibrium Point of the Model

The disease (COVID-19)-free equilibrium point *E*_0_ of model system (1) is calculated by equating all the right hand side equations to zero and putting *E*_*h*(*t*)_ = *I*_*h*(*t*)_ = 0; then, we obtained

(7) $$\begin{array}{c} E_{0}=\left(S_{h}^{0},A_{h}^{0},U_{h}^{0},E_{h}^{0},I_{h}^{0},R_{h}^{0}\right)=\left(\frac{\Lambda_{h}}{\alpha_{h}+\phi_{h}},\frac{\left(1-\theta \right)\phi_{h}\Lambda_{h}}{\alpha_{h}\left(\alpha_{h}+\phi_{h}\right)},\frac{\theta \phi_{h}\Lambda_{h}}{\alpha_{h}\left(\alpha_{h}+\phi_{h}\right)},0,0,0\right). \end{array}$$

In epidemiological point of view, the implication of *E*_0_ in the absence of infective populations and immigrants of COVID-19 asymptomatic humans only, susceptible, aware susceptible, and unaware susceptible class of human populations will live in the community.

### 3.2. Basic Reproduction Number of the Model

In this subsection, we will find the basic reproduction number, denoted by *R*_0_ for the considered model (1) using next generation approach [23]. From model system (1), matrix that consists the rate of new infections *ℱ*_*i*_(*x*) and the rate of transfer *𝒱*_*i*_(*x*) is

(8) $$\begin{array}{c} F_{i}\left(x\right)=\left[\begin{array}{c} \;\beta \left(\alpha_{2}E_{h}+\alpha_{1}I_{h}\right)U_{h}\\ 0 \end{array}\;\right],\\ V_{i}\left(x\right)=\left[\begin{array}{c} \left(\alpha_{h}+\delta_{h}-\eta_{h}\right)E_{\text{h}}\\ -\tau \delta_{h}E_{h}+\left(\alpha_{h}+\rho_{h}+\gamma_{h}\right)I_{\text{h}} \end{array}\right], \end{array}$$

respectively.

The Jacobian matrix of *ℱ*_*i*_(*x*) and *𝒱*_*i*_(*x*) at the disease-free equilibrium point *E*_0_ is given by *F* and *V*, respectively, as follows:

(9) $$\begin{array}{c} F=\left[\begin{array}{cc} \beta \alpha_{2}U_{h}^{0} & {\beta \alpha }_{1}U_{h}^{0}\\ 0 & 0 \end{array}\right],V=\left[\begin{array}{cc} \alpha_{h}+\delta_{h}-\eta_{h} & 0\\ -\tau \delta_{h} & \alpha_{h}+\rho_{h}+\gamma_{h} \end{array}\right], \end{array}$$

and then, the inverse of *V* is given by

(10) $$\begin{array}{c} V^{-1}=\left[\begin{array}{cc} \frac{1}{\alpha_{h}+\delta_{h}-\eta_{h}}\; & 0\\ \frac{\tau \delta_{h}}{\left(\alpha_{h}+\delta_{h}-\eta_{h}\right)\left(\alpha_{h}+\rho_{h}+\gamma_{h}\right)} & \frac{1}{\alpha_{h}+\rho_{h}+\gamma_{h}} \end{array}\right]. \end{array}$$

Finally, $FV^{-1}=\left[\begin{array}{cc} \left(\Lambda_{h}\beta \theta \phi_{h}\left(\alpha_{2}\left(\alpha_{h}+\phi_{h}\right)+\alpha_{1}\tau \delta_{h}\right)/\left.\alpha_{h\;\alpha_{h}+\phi_{h}}\right)\left(\alpha_{h}+\delta_{h}-\eta_{h}\right)\left(\alpha_{h}+\rho_{h}+\gamma_{h}\right)\right)\; & \Lambda_{h}\beta \theta \phi_{h}\alpha_{1}/\alpha_{h}\left(\alpha_{h}+\phi_{h}\right)\left(\alpha_{h}+\rho_{h}+\gamma_{h}\right)\\ 0 & 0 \end{array}\right]$.

The two eigenvalues of *FV*^−1^ are as follows:

(11) $$\begin{array}{c} \lambda_{1}=0,\\ \lambda_{2}=\frac{\Lambda_{h}\beta \theta \phi_{h}\left(\alpha_{2}\left(\alpha_{h}+\phi_{h}\right)+\alpha_{1}\tau \delta_{h}\right)}{\alpha_{h}\left(\alpha_{h}+\phi_{h}\right)\left(\alpha_{h}+\delta_{h}-\eta_{h}\right)\left(\alpha_{h}+\rho_{h}+\gamma_{h}\right)}. \end{array}$$

It follows that

(12) $$\begin{array}{c} R_{0}=\max\left\{\lambda_{1},\lambda_{2}\right\}=\frac{\Lambda_{h}\beta \theta \phi_{h}\left(\alpha_{2}\left(\alpha_{h}+\phi_{h}\right)+\alpha_{1}\tau \delta_{h}\right)}{\alpha_{h}\left(\alpha_{h}+\phi_{h}\right)\left(\alpha_{h}+\delta_{h}-\eta_{h}\right)\left(\alpha_{h}+\rho_{h}+\gamma_{h}\right)}. \end{array}$$

Here, 1/(*α*_*h*_ + *δ*_*h*_ − *η*_*h*_)  refers the average duration of human population in exposed state to become infectious or recovered, and 1/(*α*_*h*_ + *ρ*_*h*_ + *γ*_*h*_)  is the average duration of the infectious period of human populations until they die or recover.

### 3.3. Stability Behavior of the COVID-19-Free Equilibrium Point

The following theorems discuss the local and global stability analysis of disease-free equilibrium point of model system (1).

**Theorem 3 .**

The disease-free equilibrium point *E*_0_ of model system (1) is locally asymptotically stable if *R*_0_ < 1 and unstable if *R*_0_ > 1.

**Proof**

The Jacobian matrix of system (1) at the disease-free equilibrium *E*_0_ is given by

(13) $$\begin{array}{c} J\left(E_{0}\right)=\left[\begin{array}{cccccc} -\left(\alpha_{h}+\phi_{h}\right) & 0 & 0 & 0 & 0 & 0\\ \left(1-\theta \right)\phi_{h}\; & -\alpha_{h} & 0 & 0 & 0 & \omega_{h}\\ \;\theta \phi_{h} & 0 & -\alpha_{h} & -\frac{\beta \alpha_{2}\theta \phi_{h}\Lambda_{h}}{\alpha_{h}\left(\alpha_{h}+\phi_{h}\right)}\; & -\frac{\theta \beta \alpha_{1}\phi_{h}\Lambda_{h}}{\alpha_{h}\left(\alpha_{h}+\phi_{h}\right)} & 0\\ 0 & 0 & 0\; & \;\frac{\beta \alpha_{2}\theta \phi_{h}\Lambda_{h}}{\alpha_{h}\left(\alpha_{h}+\phi_{h}\right)}-\left(\alpha_{h}+\delta_{h}-\eta_{h}\right) & \frac{\beta \alpha_{1}\theta \phi_{h}\Lambda_{h}}{\alpha_{h}\left(\alpha_{h}+\phi_{h}\right)} & 0\\ 0 & 0 & 0 & \tau \delta_{h} & -\left(\alpha_{h}+\rho_{h}+\gamma_{h}\right) & 0\\ 0 & 0 & 0 & \left(1-\tau \right)\delta_{h} & \gamma_{h} & -\left(\alpha_{h}+\omega_{h}\right) \end{array}\right], \end{array}$$

and the characteristic polynomial of *J*(*E*_0_) is expressed by the following:

(14) $$\begin{array}{c} P\left(\lambda \right)=\left(\lambda +\left(\alpha_{h}+\phi_{h}\right)\right){\left(\lambda +\alpha_{h}\right)}^{2}\left(\lambda +\left(\alpha_{h}+\omega_{h}\;\right)\right)\left[A_{0}\lambda^{2}+A_{1}\lambda +{\text{A}}_{2}\right]=0. \end{array}$$

The four eigenvalues of *J*(*E*_0_) are as follows:

*λ*_1_ = −(*α*_*h*_ + *ϕ*_*h*_) < 0, *λ*_2_ = *λ*_3_ = −*α*_*h*_ < 0, and *λ*_4_ = −(*α*_*h*_ + *ω*_*h*_) < 0, and the remaining eigenvalues are determined from *k*(*λ*) = *A*_0_*λ*^2^ + *A*_1_*λ* + *A*_2_ = 0, where

(15) $$\begin{array}{c} A_{0}=1>0,\\ A_{1}=\left(\alpha_{h}+\rho_{h}+\gamma_{h}\right)\left(1-\frac{\beta \alpha_{2}\theta \phi_{h}\Lambda_{h}}{\alpha_{h}\left(\alpha_{h}+\phi_{h}\right)\left(\alpha_{h}+\rho_{h}+\gamma_{h}\;\right)}\right)+\left(\alpha_{h}+\delta_{h}-\eta_{h}\right),\\ A_{2}=\left(\alpha_{h}+\rho_{h}+\gamma_{h}\right)\left(\alpha_{h}+\delta_{h}-\eta_{h}\right)\left(1-\frac{\Lambda_{h}\beta \theta \phi_{h}\left(\alpha_{2}\left(\alpha_{h}+\phi_{h}\right)+\alpha_{1}\tau \delta_{h}\right)}{\alpha_{h}\left(\alpha_{h}+\phi_{h}\right)\left(\alpha_{h}+\delta_{h}-\eta_{h}\right)\left(\alpha_{h}+\rho_{h}+\gamma_{h}\right)}\right)=\left(\alpha_{h}+\rho_{h}+\gamma_{h}\right)\left(\alpha_{h}+\delta_{h}-\eta_{h}\right)\left(1-R_{0}\right). \end{array}$$

From *A*_0_*λ*^2^ + *A*_1_*λ* + *A*_2_ = 0, we recall Routh-Hurwitz criteria [24, 25] and characteristic equation *A*_0_*λ*^2^ + *A*_1_*λ* + *A*_2_ = 0 has strictly negative real root if and only if *A*_0_ > 0, *A*_1_ > 0, *A*_2_ > 0, and *A*_1_*A*_2_ > 0. It is obvious that *A*_0_ and *A*_1_ are positive, and *A*_2_ is positive provided that  1 − *R*_0_ > 0, which leads to *R*_0_ < 1. Therefore, the disease-free equilibrium point *E*_0_  is locally asymptotically stable if *R*_0_ < 11, and COVID-19 cannot invade the population. For  *R*_0_ > 1, we see that  *A*_2_ < 0. This shows as there is one eigenvalue with positive real part, and hence, the disease-free equilibrium is unstable and the invasion of COVID-19 is always possible.

**Theorem 4 .**

The COVID-19-free equilibrium point *E*_0_ of model system of equation (1) is globally asymptotically stable if *R*_0_ < 1 and vice versa.

**Proof**

To prove this, we follow Castillo-Chavez et al. theorem [26].

Let us rewrite model system (1)

(16) $$\begin{array}{c} \frac{dX}{dt}=F\left(X,Z\right),\\ dZ/dt=G\left(X,Z\right), \end{array}$$

with *G*(*X*, 0) = 0, where *X* = (*S*_*h*_(*t*), *A*_*h*_(*t*), *U*_*h*_(*t*), *R*_*h*_(*t*)) ∈ ℝ^4^ represents nondisease state variables and *X* = (*E*_*h*_(*t*), *I*_*h*_(*t*)) ∈ ℝ^2^ represents disease state variables in model (1).

To be *E*_0_ globally asymptotically stable for model system (1), the following two scenarios must satisfied:

(*H*_1_) For *dX*/*dt* = *F*(*X*, 0), *X*^∗^ is globally asymptotically stable, where *F*(*X*^∗^, 0) = 0.

Clearly one can see that at disease-free equilibrium point *E*_0_ of model system (1),

(17) $$\begin{array}{c} \underset{t⟶\infty }{\text{Lim}}\left(S_{h}\left(t\right),A_{h}\left(t\right),U_{h}\left(t\right),R_{h}\left(t\right)\right)=\left(\frac{\Lambda_{h}}{\alpha_{h}+\phi_{h}},\frac{\left(1-\theta \right)\phi_{h}\Lambda_{h}}{\alpha_{h}\left(\alpha_{h}+\phi_{h}\right)},\frac{\theta \phi_{h}\Lambda_{h}}{\alpha_{h}\left(\alpha_{h}+\phi_{h}\right)},0\right). \end{array}$$

Hence, (*S*_*h*_(*t*), *A*_*h*_(*t*), *U*_*h*_(*t*), *R*_*h*_(*t*))⟶(*Λ*_*h*_/*α*_*h*_ + *ϕ*_*h*_, (1 − *θ*)*ϕ*_*h*_*Λ*_*h*_/*α*_*h*_(*α*_*h*_ + *ϕ*_*h*_), *θϕ*_*h*_*Λ*_*h*_/*α*_*h*_(*α*_*h*_ + *ϕ*_*h*_), 0) which implies the global convergence of model system (1) in *Σ*.

$\left(H_{2}\right)G\left(X,Z\right)=AZ-\overset{\wedge }{G}\left(X,Z\right)$ , $\overset{\wedge }{G}\left(X,Z\right)\geq 0$ for (*X*, *Z*) ∈ *Σ*, where *A* = *∂Z*(*E*_0_)/*∂*(*E*_*h*_, *I*_*h*_), *Z* = (*E*_*h*_, *I*_*h*_)^*T*^, and $G\left(X,Z\right)={\left(\dot{E_{h}},{\dot{I}}_{h}\right)}^{T}$. Thus,

(18) $$\begin{array}{c} A=\left[\begin{array}{cc} \;\frac{\beta \alpha_{2}\theta \phi_{h}\Lambda_{h}}{\alpha_{h}\left(\alpha_{h}+\phi_{h}\right)}-\left(\alpha_{h}+\delta_{h}-\eta_{h}\right) & \frac{\beta \alpha_{1}\theta \phi_{h}\Lambda_{h}}{\alpha_{h}\left(\alpha_{h}+\phi_{h}\right)}\\ \tau \delta_{h} & -\left(\alpha_{h}+\rho_{h}+\gamma_{h}\right) \end{array}\right]. \end{array}$$

From $G\left(X,Z\right)=AZ-\overset{\wedge }{G}\left(X,Z\right)$, we have that $\overset{\wedge }{G}\left(X,Z\right)=AZ-G\left(X,Z\right)$, and after simplification, we get that

(19) $$\begin{array}{c} \overset{\wedge }{G}\left(X,Z\right)=\left[\begin{array}{c} \frac{\beta \theta \phi_{h}\Lambda_{h}\left(\alpha_{2}E_{h}+\alpha_{1}I_{h}\right)}{\alpha_{h}\left(\alpha_{h}+\phi_{h}\right)}-\beta \left(\alpha_{2}E_{h}+\alpha_{1}I_{h}\right)U_{h}\\ 0 \end{array}\right]. \end{array}$$

Clearly *A* is an *M*-matrix (off diagonal elements of *A* are nonnegative inside *Σ*) and $\overset{\wedge }{G}\left(X,Z\right)\geq 0$ because *U*_*h*_(*t*) = *θϕ*_*h*_[*Λ*_*h*_/*α*_*h*_ + *ϕ*_*h*_ + (*S*_*h*_(0) − *Λ*_*h*_/*α*_*h*_ + *ϕ*_*h*_)*e*^−(*α*_*h*_ + *ϕ*_*h*_)*t*^ ] ≤ *βθϕ*_*h*_*Λ*_*h*_/*α*_*h*_(*α*_*h*_ + *ϕ*_*h*_) as *t*⟶∞.

Therefore, the disease-free equilibrium point *E*_0_is globally asymptotically stable for model equation (1) when *R*_0_ < 1, and the epidemiological implication of this result is that in the long run, the disease (COVID-19) will die out in the population if the awareness is highly created in the population and in the absence of COVID-19 asymptomatic human immigrants regardless of infective populations within the community.

### 3.4. Existence and Local Stability of COVID-19 Persistent Steady State

Let *E*^∗^ = ( *S*_*h*,_^∗^*A*_*h*_^∗^, *U*_*h*_^∗^, *E*_*h*_^∗^, *I*_*h*_^∗^, *R*_*h*_^∗^)  be COVID-19 persistent equilibrium point of model equation (1) in which all state variables are to be positive, and hence, the disease (COVID-19) persists in the population. The components of *E*^∗^ are obtained by making the right hand sides of model equation (1) to be zeros, and after some mathematical manipulation we get the following:

(20) $$\begin{array}{c} S_{h}^{*}=\frac{\Lambda_{h}}{\alpha_{h}+\phi_{h}},\\ U_{h}^{*}=\frac{\left(\alpha_{h}+\delta_{h}-\eta_{h}\right)E_{h}^{*}\;}{\alpha_{h}\left(R_{0}-1\right)},\\ E_{h}^{*}=\frac{\alpha_{h}\;\left(\alpha_{h}+\rho_{h}+\gamma_{h}\;\right)\;\left(R_{0}-1\right)\;}{\beta \alpha_{1}\tau \delta_{h}+\beta \alpha_{2}\left(\alpha_{h}+\rho_{h}+\gamma_{h}\right)},\\ I_{h}^{*}=\frac{\tau \delta_{h}E_{h}^{*}}{\left(\alpha_{h}+\rho_{h}+\gamma_{h}\right)},\\ R_{h}^{*}=\frac{\left(\gamma_{h}\tau \delta_{h}+\left(\alpha_{h}+\rho_{h}+\gamma_{h}\right)\left(1-\tau \right)\delta_{h}\right)E_{h}^{*}\;}{\left(\alpha_{h}+\omega_{h}\right)\left(\alpha_{h}+\rho_{h}+\gamma_{h}\right)},\\ A_{h}^{*}=\frac{\left(\gamma_{h}\tau \delta_{h}+\left(\alpha_{h}+\rho_{h}+\gamma_{h}\;\right)\left(\alpha_{h}+\omega_{h}\right)\left(1-\theta \right)\phi_{h}\Lambda_{h}+\gamma_{h}\tau \delta_{h}+\left(1-\tau \right)\omega_{h}\delta_{h}\right){ME}_{h}^{*}\;}{\alpha_{h}\left(\alpha_{h}+\omega_{h}\right)\left(\alpha_{h}+\rho_{h}+\gamma_{h}\right)\left(\alpha_{h}+\phi_{h}\right)}, \end{array}$$

where

(21) $$\begin{array}{c} M=\left(\alpha_{h}+\rho_{h}+\gamma_{h}\right)\left(\alpha_{h}+\text{x}\phi_{h}\right). \end{array}$$

As we observe, *S*_*h*_^∗^ is positive and from the value of *E*_*h*_^∗^, it is obvious that all the values of ( *A*_*h*_^∗^, *U*_*h*_^∗^, *I*_*h*_^∗^, *R*_*h*_^∗^) are positive for *R*_0_ > 1.

**Corollary 1 .**

The COVID-19 persistent equilibrium point *E*^∗^ of model system (1) exists only when  *R*_0_ > 1.

**Theorem 5 .**

The COVID-19 persistent steady state *E*^∗^ = (*S*_*h*_^∗^,*A*_*h*_^∗^,*U*_*t*_^∗^,*E*_*h*_^∗^,*I*_*h*_^∗^,*R*_*h*_^∗^) of model (1) is locally asymptotically stable if and only if *R*_0_ > 1.

**Proof**

The linearized matrix of the model system (1) at the endemic steady state *E*^∗^ is given by

(22) $$\begin{array}{c} J\left(E^{*}\right)=\left[\begin{array}{cccccc} -k_{1} & 0 & 0 & 0 & 0 & 0\\ k_{2}\; & -\alpha_{h} & 0 & 0 & 0 & \omega_{h}\\ \;k_{3} & 0 & -\left(k_{6}E_{h}^{*}+k_{4}I_{h}^{*}+\alpha_{h}\right) & -k_{6}U_{h}^{*}\; & -k_{4}U_{h}^{*} & 0\\ 0 & 0 & k_{6}E_{h}^{*}+k_{4}I_{h}^{*}\; & \;k_{6}U_{h}^{*}-\left(\alpha_{h}+\delta_{h}-\eta_{h}\right) & \;k_{4}U_{h}^{*} & 0\\ 0 & 0 & 0 & k_{7} & -k_{8} & 0\\ 0 & 0 & 0 & k_{9} & \gamma_{h} & -k_{10} \end{array}\right], \end{array}$$

where

(23) $$\begin{array}{c} k_{1}=\left(\alpha_{h}+\phi_{h}\right),k_{2}=\left(1-\theta \right)\phi_{h},k_{3}=\theta \phi_{h},k_{4}=\alpha_{1}\beta ,k_{5}=\phi_{h}+\delta_{h},k_{6}=\beta \alpha_{2},k_{7}=\tau \delta_{h},k_{8}=\alpha_{h}+\rho_{h}+\gamma_{h},k_{9}=\left(1-\tau \right)\delta_{h},k_{10}=\alpha_{h}+\omega_{h},A=k_{4}k_{7}+k_{6}k_{8}. \end{array}$$

The three eigenvalues of *J*(*E*^∗^) are *λ*_1_ = −*k*_1_ = −(*α*_*h*_ + *ϕ*_*h*_) < 0, *λ*_2_ = −*α*_*h*_ < 0, *λ*_3_ = −*k*_10_ = −(*α*_*h*_ + *ϕ*_*h*_) < 0, and the remaining eigenvalues are determined from the submatrix given by

(24) $$\begin{array}{c} J_{4}\left(E^{*}\right)=\left[\;\begin{array}{ccc} \frac{-\left(k_{6}k_{8}\;E_{h}^{*}+\alpha_{h}k_{8}+k_{4}k_{7}\;E_{h}^{*}\right)}{k_{8}}\; & \frac{-k_{6}\left(k_{5}-\eta_{h}\right)E_{h}^{*}}{\alpha_{h}\left(R_{0}-1\right)} & \frac{-k_{4}\left(k_{5}-\eta_{h}\right)E_{h}^{*}}{\alpha_{h}\left(R_{0}-1\right)}\\ \frac{\left(k_{6}k_{8}\;E_{h}^{*}+k_{4}k_{7}\;E_{h}^{*}\right)}{k_{8}}\; & \frac{k_{6}\left(k_{5}-\eta_{h}\right)E_{h}^{*}}{\alpha_{h}\left(R_{0}-1\right)} & \frac{k_{4}\left(k_{5}-\eta_{h}\right)E_{h}^{*}}{\alpha_{h}\left(R_{0}-1\right)}\\ 0 & k_{7} & -k_{8} \end{array}\right]. \end{array}$$

From COVID-19 persistent equilibrium point *E*^∗^, further substitution of the value of *k* and *A*, we get that

(25) $$\begin{array}{c} E_{h}^{*}=\frac{\alpha_{h}k_{8}\left(R_{0}-1\right)}{k_{4}k_{7}+k_{6}k_{8}}=\frac{\alpha_{h}k_{8}\left(R_{0}-1\right)}{\text{A}},U_{h}^{*}=\frac{k_{8}\left(k_{5}-\eta_{h}\right)E_{h}^{*}}{k_{4}k_{7}+k_{6}k_{8}}=\frac{k_{8}\left(k_{5}-\eta_{h}\right)E_{h}^{*}}{\text{A}}\text{and }I_{h}^{*}=\frac{k_{7}E_{h}^{*}}{k_{8.}}. \end{array}$$

Now without calculating the eigenvalues of  *J*_4_(*E*^∗^), we can simply look the signs of its eigenvalues by using trace determinant rule as follows: trace( *J*_4_(*E*^∗^)) = −[*α*_*h*_*R*_0_*A* + *k*_4_*k*_7_(*k*_5_ − *η*_*h*_) + *k*_8_*A*]/*A* < 0. Therefore, trace( *J*_4_(*E*^∗^)) < 0. Furthermore, det( *J*_4_*E*^∗^) = *k*_6_*k*_8_^2^*α*_*h*_[(*k*_5_ − *η*_*h*_)*R*_0_ − (*R*_0_ − 1)]/*A* + *α*_*h*_(*k*_5_ − *η*_*h*_)[*k*_4_*R*_0_ + *k*_6_*k*_8_*R*_0_ + *k*_4_*k*_7_]/*A* > 0, which can be verified by substitute trace( *J*_4_(*E*^∗^)) inequality into det ( *J*_4_(*E*^∗^)). Thus, the above scenarios, i.e., *λ*_1_ = −*k*_1_ = −(*α*_*h*_ + *ϕ*_*h*_) < 0,  *λ*_2_ = −*α*_*h*_ < 0,  *λ*_3_ = −*k*_10_ = −(*α*_*h*_ + *ϕ*_*h*_) < 0, and trace( *J*_4_(*E*^∗^)) < 0 and det ( *J*_4_(*E*^∗^)) > 0, lead to the COVID-19 persistent steady state *E*^∗^ of model system (1) which is locally asymptotically stable whenever *R*_0_ > 1. Hence, it is the required result.

### 3.5. Sensitivity Analysis of the Basic Reproduction Number

Sensitivity analysis helps to identify the most influential parameters on the basic reproduction number so efforts to control the problem are directed to these parameters. Mathematically, we compute the sensitivity analysis of our model system of equations based on the classical definition [14, 15] defined as the normalized forward sensitivity index of a variable *R*_0_, which depends differentiably on a parameter *p*, given by △_*p*_^*R*_0_^ = *p*/*R*_0_ × *∂R*_0_/*∂p*.

For example, △_*β*_^*R*_0_^ ≡ 1, means increasing (or decreasing) the contact rate *β* of unaware susceptible with exposed and infectious individuals by 10%, will result to increase (or decrease) the value of *R*_0_ by 10%, whereas △_*δ*_*h*__^*R*_0_^≡−5.11, means increasing (or decreasing) the recovery rate of infectious humans, will result to decrease (or increase) the value of *R*_0_ by 51.1%. The remaining sensitivity analysis of our model can be obtained as follows:

(26) $$\begin{array}{c} {△}_{\beta }^{R_{0}}=1>0,{△}_{\alpha_{1}}^{R_{0}}=\frac{\alpha_{1}\tau \delta_{h}}{\alpha_{1}\tau \delta_{h}+\alpha_{h}\left(\alpha_{h}+\rho_{h}+\gamma_{h}\right)}>0,{△}_{\tau }^{R_{0}}=\frac{\alpha_{1}\tau \delta_{h}}{\alpha_{1}\tau \delta_{h}+\alpha_{2}\left(\alpha_{h}+\rho_{h}+\gamma_{h}\right)}>0,\\ {△}_{\phi_{h}}^{R_{0}}=\frac{\alpha_{h}}{\left(\alpha_{h}+\phi_{h}\right)}>0,{△}_{\theta }^{R_{0}}=1>0,{△}_{\alpha_{2}}^{R_{0}}=\frac{\;\alpha_{2}\left(\alpha_{h}+\rho_{h}+\gamma_{h}\right)}{\alpha_{1}\tau \delta_{h}+\alpha_{2}\left(\alpha_{h}+\rho_{h}+\gamma_{h}\right)}>0,{△}_{\eta_{h}}^{R_{0}}=\frac{\eta_{h}}{\alpha_{1}+\delta_{h}-\eta_{h}}>0,\\ {△}_{\gamma_{h}}^{R_{0}}=\frac{\;\alpha_{2}\gamma_{h}}{\alpha_{1}\tau \delta_{h}+\alpha_{2}\left(\alpha_{h}+\rho_{h}+\gamma_{h}\right)}-\frac{1}{\left(\alpha_{h}+\rho_{h}+\gamma_{h}\right)}<0,{△}_{\delta_{h}}^{R_{0}}=\frac{\;\delta_{h}\tau \alpha_{1}}{\alpha_{1}\tau \delta_{h}+\alpha_{2}\left(\alpha_{h}+\rho_{h}+\gamma_{h}\right)}-\frac{1}{\alpha_{1}+\delta_{h}-\eta_{h}}<0. \end{array}$$

The sensitivity analysis indices evaluated at the baseline model parameters values are resembled from Table 2. The sensitivity indices are arranged in descending order as follows.

#### 3.5.1. Interpretation of Sensitivity Indices

From Table 2, parameters that have positive indices have a negative impact in the control of COVID-19 transmission if their values are increasing. On the other hand, parameters which have negative indices have a positive impact to minimize the burden of the disease (COVID-19) transmission in the society. Thus, the most sensitive parameters for the transmission of disease (COVID-19) are *β* and *θ*, and the most sensitive parameter for the control of the disease (COVID-19) is *δ*_*h*_  followed by *γ*_*h*_.

## 4. Numerical Results and Discussion

### 4.1. Graphs for General Population Dynamics

In this section, to verify the theoretical results of the model, numerical simulations are carried out by using MATLAB ode45 solver with the following initial conditions:

*S*_*h*0_ = 120,000, *A*_*h*0_ = 30,000, *U*_*h*0_ = 50,000, *E*_*h*0_ = 25,000, *I*_*h*0_ = 35,000, *R*_*h*0_ = 20,000. It is important to note that parameter values and initial number of populations are taken for illustrative purpose.

**Figure 2:**
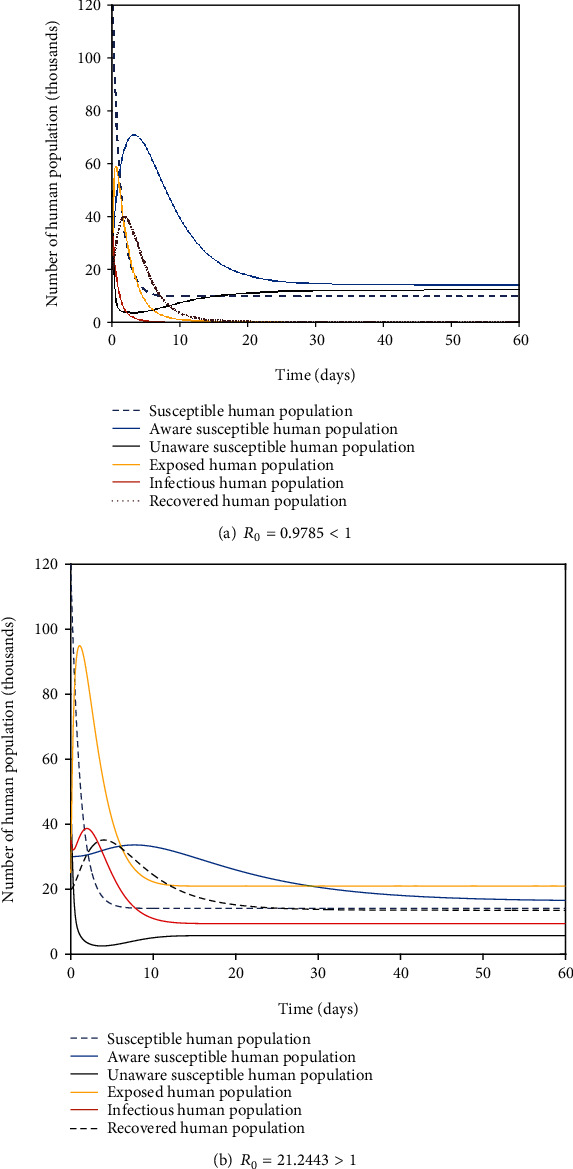
Change of human populations at disease free and endemic equilibrium points.

Figure 2(a)reflects that when the value of *Λ*_*h*_ = 7.99, *θ* = 0.47,  *α*_1_ = 0.19,  *α*_2_ = 0.1399,  *α*_*h*_ = 0.22,  *ϕ*_*h*_ = 0.59,  *ω*_*h*_ = 0.1,  *τ* = 0.09,  *β* = 0.389,  *δ*_*h*_ = 0.6, *ρ*_*h*_ = 0.99, *γ*_*h*_ = 0.1, and *η*_*h*_ = 0.0898, then  *R*_0_ = 0.9785 < 1. It is shown that all trajectories of the solutions of the model system (1) converge towards disease-free equilibrium point components, or noninfective class of human population tends to nonzero components, and the infective class of human population tends to zero component. In this case, the basic reproduction number is less than unity (*R*_0_ = 0.9785 < 1) which confirms with our local stability analysis of disease (COVID-19)-free equilibrium point for model system (1) whenever *R*_0_ < 1  stated from Theorem 4.

Figure 2(b)reflects that when parameter values changed as *Λ*_*h*_ = 12.4, *θ* = 0.99, *α*_1_ = 0.2,  *α*_2_ = 0.14,  *α*_*h*_ = 0.0701,  *ϕ*_*h*_ = 0.79,  *ω*_*h*_ = 0.1,  *τ* = 0.79,  *β* = 0.39,  *δ*_*h*_ = 0.5, *ρ*_*h*_ = 0.75, *γ*_*h*_ = 0.0391, and *η*_*h*_ = 0.0899, then *R*_0_ = 21.2443 > 1. It is shown that all trajectories of solutions of model system (1) converge towards the endemic (COVID-19 persistent) equilibrium point components of model (1), or all distinct classes of human populations coexist. In this case, the basic reproduction number is greater than unity (*R*_0_ = 21.2443 > 1) which supports our analytical result about local stability of endemic equilibrium point *E*^∗^ for model system (1) whenever *R*_0_ > 1 stated from Theorem 7.

### 4.2. Graphs on Newly Included Parameters in the Model

**Figure 3:**
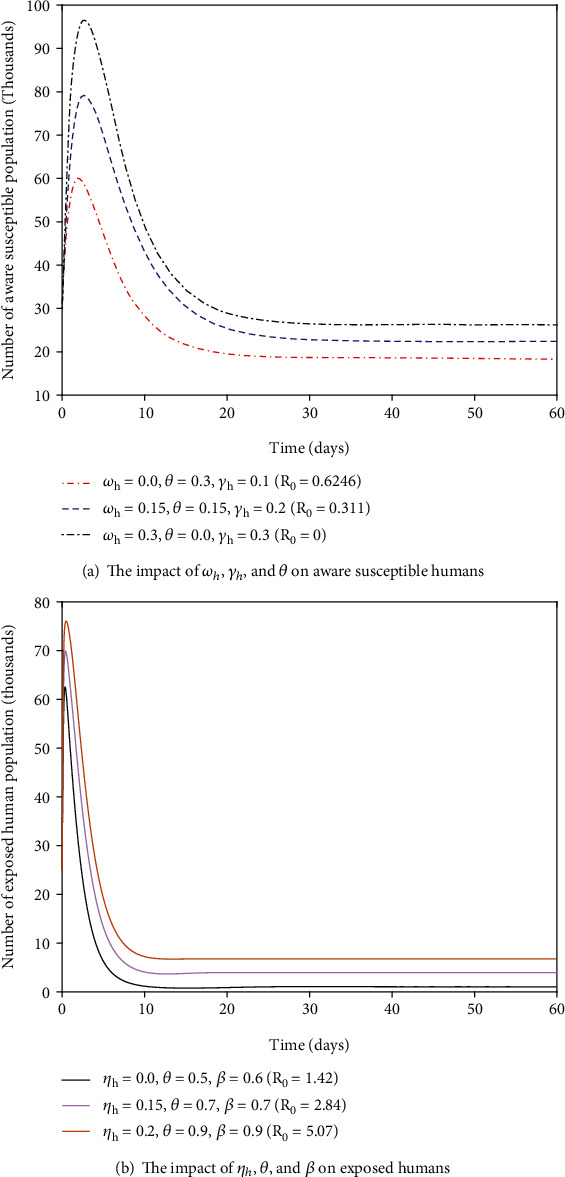
The impacts of some parameters on aware susceptible and exposed human populations.

As we observe from Figure 3(a), when the values of *ω*_*h*_  and *γ*_*h*_ increase and the values of *θ* decreases while other parameter values remain constant, then the value of the secondary infection, *R*_0_, decreases and this leads to increase the number of aware susceptible human population. If we implement effective awareness creation mechanisms between individuals, then the transmission rate and the spread of (COVID-19) will be eliminated. As we observe from Figure 3(b), when the values of *η*_*h*_, *θ*, and *β* increase, then the values of secondary infection, *R*_0_, increases. If we implement effective mechanisms to create awareness between humans to avoid contacts and reduce the immigration rate of COVID-19 asymptomatic humans, then the number of exposed human population will be reduced and also the transmission of COVID-19 pandemic will be reduced.

### 4.3. Graphs for Sensitive Analysis of the Model

**Figure 4:**
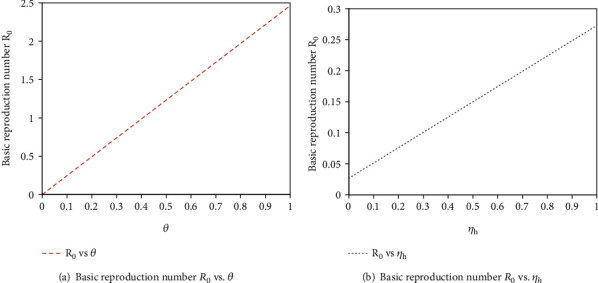
The impact of *θ* and *η*_*h*_  on basic reproduction number *R*_0_.

**Figure 5:**
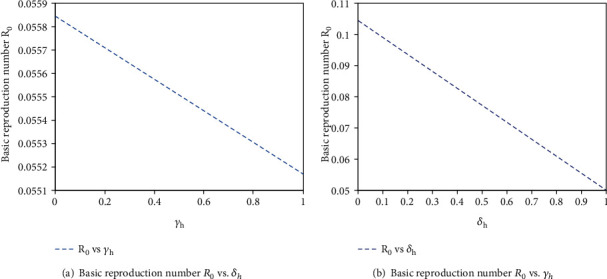
The impact of *δ*_*h*_ and *γ*_*h*_  on basic reproduction number *R*_0_.

The authors can find some significant results which have shown in Figures 4(a) and 4(b), and one can observe that the large value of *θ* or *η*_*h*_ can lead to the large value of secondary infection *R*_0_. This implies that high proportion rate from susceptible human into unaware susceptible human or high rate of COVID-19 asymptomatic human immigrants can increase the opportunity of COVID-19 outbreak. Generally, from Figure 4, we found that *R*_0_ is more sensitive to the proportion rate (*θ*) of susceptible humans into unaware susceptible human than the rate of COVID-19 asymptomatic human immigrant (*η*_*h*_). This supports the idea that *θ* is the most sensitive parameter in the transmission of COVID-19. Therefore, decreasing the proportion rate of humans into unaware human class by creating unlimited awareness between individuals helps to reduce COVID-19 outbreak. From Figures 5(a) and 5(b), we can see that the large value of *δ*_*h*_ or *γ*_*h*_ can lead to the small value of secondary infection, *R*_0_. This is to say that the high progression rate from exposed into infectious or recovered class and the recovery rate from infectious human class into recovered human class can decrease the opportunity of COVID-19 outbreak. Generally, from Figure 5, we found that *R*_0_ is less sensitive to the progression rate (*δ*_*h*_) of exposed humans into infectious and recovered human class than the recovery rate *γ*_*h*_ of individuals from their infection. This supports the idea of sensitivity analysis result that *δ*_*h*_  is the most sensitive parameter in the control of COVID-19 transmission than  *γ*_*h*_.

**Figure 6:**
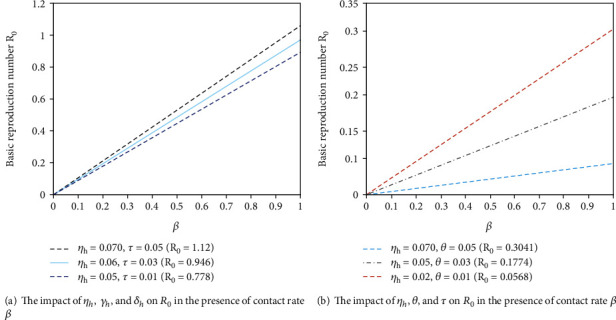
The impact of on *η*_*h*_, *γ*_*h*_,  *δ*_*h*_,  *θ*, and *τ*  on *R*_0_ in the presence of contact rate *β*.

In Figure 6(a), it can be seen that large value of *δ*_*h*_ or *γ*_*h*_  and small value of *η*_*h*_ in the presence of *β* can lead to small value of *R*_0_. That is to say, if we increase the progression rate of exposed into infectious by diagnosis to be quarantined or recovered human class by treatment and the rate of COVID-19 asymptomatic humans into exposed human class by prediagnosis in the presence of human to human contact, the transmission of COVID-19 pandemic will decrease in the population. From Figure 6(b), it can be seen that large value of *θ* or *η*_*h*_ or *τ*  can lead to the large value of *R*_0_. If we reduce the proportion rate from susceptible human class into unaware susceptible human class by creating awareness and the rate of infective COVID-19 asymptomatic human immigrants by effective prediagnosis, then the disease (COVID-19) outbreak will end.

## 5. Conclusion

In this study, a nonlinear deterministic mathematical model of COVID-19 pandemic is developed and analyzed to investigate the impact of awareness and COVID-19 asymptomatic human immigrants in the transmission of COVID-19. We first obtained the domain where the model gives epidemiologically meaningful and mathematically well-posed by the fundamental existence and uniqueness theorem. Both positivity and invariant region of the model solutions are shown analytically. The basic reproduction number, *R*_0_, is computed using next generation matrix approach. The analytical analysis showed that the disease (COVID-19) free and endemic (COVID-19 persistent) equilibrium points of the model exist under certain conditions. We analyzed both the local and global stability of disease-free equilibrium point based on *R*_0_. The disease-free equilibrium point of the model is locally as well as globally asymptotically stable whenever *R*_0_ < 1 and unstable whenever *R*_0_ > 1. From epidemiological point of view, the disease (COVID-19) will die out in the population whenever <1 and persists in the population whenever *R*_0_ > 1. Positive endemic equilibrium point of the model exists, and it is locally asymptotically stable whenever *R*_0_ > 1, so that the reinvasion of COVID-19 may possible in the population.

We performed sensitivity analysis of the basic reproduction number with respect to model parameters to identify which parameters have a strong influence on COVID-19 transmission dynamical system. Both analytical analysis and numerical simulation results of the model ensured that the most sensitive parameters for the transmission of COVID-19 are *θ* in which susceptible individuals will join unaware human class and contact rate (*β*) of those unaware susceptible humans with exposed and infectious human population, while the most sensitive parameter to control COVID-19 transmission is the progression rate *δ*_*h*_ followed by the recovery rate *γ*_*h*_.

## 6. Concluding Remarks and Suggestions

It is necessary to achieve a better understanding on the COVID-19 pandemic, taking into account awareness campaign between humans and control of COVID-19 asymptomatic human immigrants in order to reduce the number of infections and mortality rates. The model developed in the present manuscript has the advantage of describing the best way of controlling the COVID-19 outbreak. As we demonstrated in the theoretical analysis and numerical results, reducing the values of *θ* (the proportion rate of susceptible into unaware human class) and *η*_*h*_ (the rate of COVID-19 asymptomatic human immigrants) helps to reduce exposed and infectious individuals. These help to control COVID-19 outbreak. Therefore, in order to control COVID-19 outbreak, policy makers or health workers must give great emphasis on how to further create awareness between humans and effective mechanisms to reduce infective COVID-19 asymptomatic human immigrants.

## Figures and Tables

**Table 1:** Model parameters with their description.

Parameter	Description of parameter

*Λ*_*h*_	The recruitment rate of susceptible human population
*β*	The contact rate of unaware susceptible humans with exposed and infectious humans
*θ*	The proportion rate that susceptible humans join unaware susceptible humans
*ϕ*_*h*_	The progression rate of susceptible into aware and unaware susceptible humans
*α*_2_	The probability that unaware susceptible humans will contact with infectious humans
*α*_1_	The probability that unaware susceptible humans will contact with exposed humans
*δ*_*h*_	The progression rate of exposed humans into infectious and recovered human class
*γ*_*h*_	The recovery rate of infectious humans into recovered human class
*ω*_*h*_	The progression rate of recovered humans into aware susceptible human class
*α*_*h*_	The natural death rate of all human population classes
*ρ*_*h*_	The disease induced death rate of infectious humans
*τ*	The proportion rate of exposed humans to be infectious
*η*_*h*_	The rate of asymptomatic human immigrants

**Table 2:** Parameter values and its sensitivity indices.

Parameter	Values	Sensitivity indices	Source
*β*	0.0143	+1	[16]
*Λ*_*h*_	13.5	+1	[16]
*θ*	0.5	+1	Assumed
*α*_2_	0.02	+0.998	[16]
*ϕ*_*h*_	0.01	+0.615	Assumed
*α*_1_	0.0001	+0.0018	[16]
*η*_*h*_	0.0001	+0.0016	Assumed
*τ*	0.7	+0.0015	[16]
*γ*_*h*_	0.15	-5.11	[16]
*δ*_*h*_	0.07	-11.64	[16]

## Data Availability

The data supporting this model are from the previous published articles and cited on a relevant places.
